# Sunitinib and bevacizumab for first-line treatment of metastatic renal cell carcinoma: a systematic review and indirect comparison of clinical effectiveness

**DOI:** 10.1038/sj.bjc.6605167

**Published:** 2009-06-30

**Authors:** J S Thompson Coon, Z Liu, M Hoyle, G Rogers, C Green, T Moxham, K Welch, K Stein

**Affiliations:** 1 Peninsula Technology Assessment Group, Peninsula Medical School, Universities of Plymouth and Exeter, Noy Scott House, Barrack Road, Exeter EX2 5DW, UK; 2 Wessex Institute, University of Southampton, Mailpoint 728, Boldrewood, Southampton SO16 7PX, UK

**Keywords:** renal cancer, indirect comparison, systematic review, sunitinib, bevacizumab

## Abstract

**Background:**

Two new agents have recently been licensed for use in the treatment of metastatic renal cell carcinoma (RCC) in Europe. This paper aims to systematically review the evidence from all available randomised clinical trials of sunitinib and bevacizumab (in combination with interferon-α (IFN-α)) in the treatment of advanced metastatic RCC.

**Methods:**

Systematic literature searches were performed in six electronic databases. Bibliographies of included studies were searched for further relevant studies. Individual conference proceedings were searched using their online interfaces. Studies were selected according to the predefined criteria. All randomised clinical trials of sunitinib or bevacizumab in combination with IFN for treating advanced metastatic RCC in accordance with the European licensed indication were included. Study selection, data extraction, validation and quality assessment were performed by two reviewers with disagreements being settled by discussion. The effects of sunitinib and bevacizumab (in combination with IFN-α) on progression-free survival were compared indirectly using Bayesian Markov Chain Monte-Carlo (MCMC) sampling in Win BUGS, with IFN as a common comparator.

**Results:**

Three studies were included. Median progression-free survival was significantly prolonged with both interventions (from approximately 5 months to between 8 and 11 months) compared with IFN. Overall survival was also prolonged, compared with IFN, although the published data are not fully mature. Indirect comparison suggests that sunitinib is superior to bevacizumab plus IFN in terms of progression-free survival (hazard ratios 0.796; 95% CI 0.63–1.0; *P*=0.0272).

**Conclusion:**

There is evidence to suggest that treatment with sunitinib and treatment with bevacizumab plus IFN has clinically relevant and statistically significant advantages over treatment with IFN alone in patients with metastatic RCC.

For the past 20 years the mainstay of treatment for people with advanced and metastatic renal cell carcinoma (RCC) has been immunotherapy (interferon-α (IFN-α) and interleukin-2). This treatment is frequently associated with unpleasant side effects and confers only modest benefits. A recently updated Cochrane Review identified 58 randomised clinical trials in which immunotherapies had been used in advanced RCC. The weighted average median survival was 3.8 months longer for IFN-α than for control treatments (11.4 *vs* 7.6 months) (Coppin *et al*, 2005). Hormonal treatments and standard chemotherapies are generally not considered effective due to high levels of drug resistance (Fojo *et al*, 1987; Mignogna *et al*, 2006). Improvements in drug treatment for metastatic RCC have therefore been a priority for pharmaceutical development, with several new-targeted agents recently becoming available. In Europe, two agents are licensed for the first-line use in metastatic RCC: sunitinib and bevacizumab (in combination with IFN-α). Sunitinib is an oral multi-targeted tyrosine kinase inhibitor that targets and blocks the signalling pathways of multiple receptor tyrosine kinases. Bevacizumab is a recombinant humanised monoclonal antibody, which inhibits angiogenesis by preventing VEGF from binding to its receptors on vascular endothelium and other vascular cells, inhibiting tumour growth and proliferation. Both drugs have orphan drug status. The aim of this review was to systematically review current evidence for the effectiveness of sunitinib and bevacizumab. As both drugs share a common indication, we considered it particularly important to investigate the comparative effectiveness of sunitinib and bevacizumab.

## Materials and methods

Systematic literature searches were performed in August 2008 in the following electronic databases: MEDLINE, Embase, Cochrane Library (2008, Issue 3) (including CDSR, CENTRAL and HTA), Science Citation Index Expanded and Web of Science Proceedings. The search strategy included terms used to describe renal cancer and the trade and proprietary names of the interventions and is available in full on request from the authors. The search was limited to original publications relating to humans. Bibliographies of included studies were searched for further relevant studies. Individual conference proceedings from the ASCO Annual Meeting (2007 and 2008), ASCO Genitourinary Cancers Symposium (2007 and 2008) and ECCO (2007) were searched on their online interface. All searches were re-run in November 2008 to identify any additional publications.

Two reviewers (JTC and ZL) independently examined titles and abstracts identified from the search and full texts of any potentially relevant studies were obtained. The relevance of each paper was then assessed independently according to the inclusion and exclusion criteria and any discrepancies were resolved by discussion.

Randomised clinical trials were included if they compared either sunitinib or bevacizumab in combination with IFN-α with standard treatment (immunotherapy or best supportive care) as first-line therapy (according to the European licensed indication) in participants with advanced and/or metastatic RCC. Primary outcomes were overall survival (OS) and progression-free survival (PFS). We also considered the adverse events and toxicity reported in the included trials. Only trials which reported at least one of the primary outcomes were included in the review. Conference abstracts were included if there was sufficient detail to assess quality or if they reported updated results of included trials.

Data were extracted by one reviewer (ZL) using a standardised data extraction form and checked independently by a second (JTC). The methodological quality of the studies was assessed using criteria specified by the Centre for Reviews and Dissemination (CRD).(NHS Centre for Reviews and Dissemination, 2001) Quality was assessed by one reviewer (ZL) and judgments checked by a second (JTC). Any disagreement was resolved by discussion, with involvement of a third reviewer (KS) as necessary.

Details of the extracted data for each individual study are presented in structured tables and as a narrative description. Survival data (OS and PFS) are presented as hazard ratios (HRs) where available.

As data on head-to-head comparisons between the interventions were not available, we performed an indirect comparison using Bayesian Markov Chain Monte-Carlo (MCMC) sampling in WinBUGS, with IFN as a common comparator (Ades, 2003), adopting a fixed-effect model. This approach assumes ‘exchangeability’ of treatment effect across all included trials, such that the observed treatment effect for any comparison could have been expected to arise if it had been measured in the populations reported in all other included trials. Exchangeability was judged through examination of the trial populations and comparability of outcomes in the common treatment group facilitating the comparison.

Vague earlier distributions were used in the analyses (*N*[0,  0.001] for log-hazard ratios). Point estimates and 95% credible intervals were calculated from 100 000 simulated draws from the posterior distribution after a burn-in of 10 000 iterations. An empirical *P*-value is also presented, simply calculated as the proportion of MCMC trials in which the HR for any given comparison exceeded 1.

## Results

### Quantity of evidence

**Figure 1:**
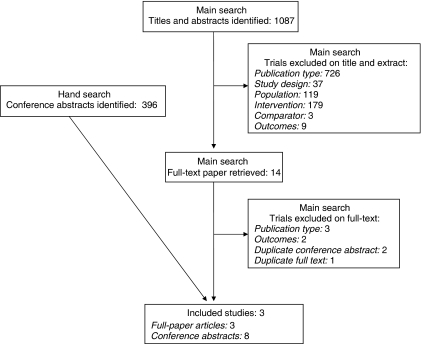
Flow chart of trial selection process.

After removal of duplicates, the initial search conducted in August 2008 generated 1087 references. One thousand and seventy three papers were excluded on title and abstract. Full-text copies of the remaining 14 citations was obtained and read in full. Six of these citations were subsequently included in the review. The majority of papers did not meet the exclusion criteria for multiple reasons; only the first identified reason was recorded. Figure 1 shows details of inclusion of papers in the systematic review.

Four additional conference abstracts were located as a result of hand searching; one of these contained duplicate data. One additional clinical trial was identified in the re-run search. A total of 11 publications were therefore included in the review; these relate to three clinical trials reported in the full-text publications (Motzer *et al*, 2007b; Escudier *et al*, 2008; Rini *et al*, 2008) and eight conference abstracts (Motzer *et al*, 2006, 2007a, 2007c; Bracarda *et al*, 2007; Escudier *et al*, 2007; Melichar *et al*, 2007; Bajetta *et al*, 2008; Figlin *et al*, 2008) relating to those trials.

Two included trials compared the effects of combination treatment with bevacizumab (10 mg kg^−1^ body weight delivered intravenously once every 2 weeks) plus IFN (nine MIU delivered subcutaneously three times per week) compared with either IFN plus placebo (AVOREN) (Escudier *et al*, 2008) or IFN alone (CALGB) (Rini *et al*, 2008) on overall and PFS, and one trial assessed the effect of sunitinib (50 mg once daily; orally in 6-week cycles – 4 weeks on and 2 weeks off) on the same outcomes (Motzer *et al*, 2007b). Study characteristics are shown in Table 1.

### Quality of evidence

All three included trials were large (>300 participants per arm), phase III, international, multi-centre, randomised clinical studies. Final OS results for the phase III trial of sunitinib *vs* IFN presented at the ASCO Annual Meeting in 2008 include one *post hoc* analysis of patients who did not receive any post-study treatment; only 47% of the original study population is included. Demographic details of the sub-group are not presented, hampering quality assessment of this analysis.

Of the included trials, one (Escudier *et al*, 2008) was placebo controlled and blinded, one trial (Motzer *et al*, 2007b) was not blinded but an independent and blinded central review of images was performed to assess PFS and objective response rate; the final trial was not blinded and progression was assessed by the investigator (Rini *et al*, 2008). All three studies were performed in similar populations – predominantly clear cell, metastatic RCC, the majority of patients having undergone nephrectomy and having favourable or intermediate prognosis and good performance status. All studies used explicit inclusion and exclusion criteria and treatment groups were well matched at study entry. None of the studies recruited patients with brain metastases (unless neurologically stable) and relatively few patients with bone metastases were included (20% in the AVOREN trial and 30% in the trial of sunitinib *vs* IFN).

### Assessment of OS

There was insufficient follow-up data to fully report median OS in any of the trials, although subsequent conference presentations have provided further details for the trial of sunitinib (Table 2) (Motzer *et al*, 2007a, 2007b, 2007c; Figlin *et al*, 2008). In the AVOREN trial, median OS in the IFN group was reported as 19.8 months and had not been reached in the bevacizumab group. The researchers provided an estimated HR of 0.79 (95% CI 0.62–1.02; *P*=0.067). No further updated results have been published for this trial. In the CALGB trial, no interim analyses of OS have yet been performed.

Although at the time of reporting the original findings (Motzer *et al*, 2007b) of the phase III trial of sunitinib *vs* IFN, median OS had not been reached in either group; the authors provided an estimated HR of 0.65 (95% CI 0.45–0.94; *P*=0.02). This comparison did not reach the pre-specified level of significance for the interim analysis. Final OS data were reported at the ASCO Annual Meeting in 2008 (Figlin *et al*, 2008). Material provided in both the ASCO abstract and presentation slides provides three analyses of OS from this study; an ITT analysis in which all patients are included, an analysis in which data from 25 patients in the IFN group who crossed over to sunitinib within the study protocol following disease progression are censored and finally a *post hoc* analysis in which only those patients (*n*=355; 47% of the total study population) who received no additional treatment following withdrawal from the study are included. All three sets of results are presented in Table 2. Estimates of median OS in patients treated with IFN range from 21.8 (95% CI 17.8–26.9) months in the final analysis to 14.1 (95% CI 9.7–21.1) months in the *post hoc* analysis. Estimates of median OS in patients treated with sunitinib also vary according to the analysis undertaken, from 26.4 (95% CI 23.9–32.9) months and 26.4 (95% CI 23.0–32.9) months in the final and censored analyses respectively to 28.1 (95% CI 19.5 to NA) in the *post hoc* analysis. That people could expect greater OS in an absence of the second-line therapy appears to be counter-intuitive, as one would expect that the additional treatment regimens would serve to prolong life. It is possible that this is a spurious result as a consequence of the small number of people in the analysis or the underlying reason for this group of people not receiving the second-line therapy, it is possible that the group of people who did not require additional treatment had a better prognosis at the start of the study or perhaps it is a reflection of a toxic effect of additional therapy. As these data are taken from a conference abstract, full details of the analysis are not available.

### Assessment of PFS

Although the method of assessment of progression varied between the trials, all three showed an improvement in median PFS compared with IFN (Table 3).

In both trials of treatment with bevacizumab plus IFN, median PFS was significantly increased compared with IFN (either alone or in combination with placebo), the effect being greater in the AVOREN (10.2 *vs* 5.4 months; HR 0.63 95% CI 0.52–0.75; *P*<0.0001) (Escudier *et al*, 2008) than the CALGB trial (8.5 *vs* 5.2 months; HR 0.71 95% CI 0.61–0.83) (Rini *et al*, 2008).

Treatment with sunitinib also improved median PFS compared with IFN treatment in all available analyses. The most complete set of data comes from an interim analysis performed in February 2007 (PFS data were not reported as part of the final analysis presented in 2008). Results from the independent central review of imaging studies showed a median PFS for patients treated with sunitinib of 11 (95% CI 10.7–13.4) months and 5.1 (95% CI 3.9–5.6) months for the IFN group, giving an HR of 0.538 (95% CI 0.439–0.658). Investigator-assessed results were similar at 10.8 (95% CI 10.6–12.6) months for sunitinib compared with 4.1 (95% CI 3.8–5.3) months for IFN producing an HR of 0.519 ((95% CI 0.435–0.618).

#### Indirect comparison of the effects of sunitinib and bevacizumab plus IFN

As we identified no evidence of head-to-head comparisons of sunitinib and bevacizumab plus IFN, we assessed the validity of performing an indirect comparison of the effects of the two interventions on OS and PFS. Examination of the key characteristics of the trials (Table 1) suggests that they are suitably similar and indicates that an indirect comparison of bevacizumab plus IFN *vs* sunitinib is appropriate. However, as there is little mature OS data available and we were unable to fully compare the trials in terms of the extent to which patients received additional medications following withdrawal, we have not performed an indirect comparison of the interventions in terms of OS.

For the indirect comparison of PFS data, we chose to use the most complete data from each trial (Table 4). The results of the indirect comparison of PFS data suggest that over the length of the assessment, people taking sunitinib were less likely to progress than those taking bevacizumab plus IFN (*P*=0.0272 (one-tailed test)). Sunitinib may therefore be superior to bevacizumab plus IFN in preventing progression in this patient population.

### Adverse events

From the adverse events reported in these trials, the safety profile of both interventions appears to be comparable with IFN, with some adverse events particularly associated with bevacizumab plus IFN (proteinuria, hypertension, bleeding events) and some with sunitinib treatment (hypertension, hand and foot syndrome and diarrhoea). The frequency of grade 3 or worse adverse events may be higher with the combination of bevacizumab and IFN than with IFN alone; in the CALGB trial 61% of people treated with IFN reported grade 3 or 4 toxicity compared with 79% treated with the combination of bevacizumab plus IFN (*P*<0.0001). During treatment with sunitinib, there were significantly fewer grade 3 or worse adverse events reported than during treatment with IFN (7 *vs* 12%; *P*<0.05).

## Discussion

Clinical data available from three randomised clinical trials indicate that treatment with sunitinib and treatment with bevacizumab (in combination with IFN) has clinically relevant and statistically significant advantages over treatment with IFN alone in terms of PFS. In the three included trials, median PFS was significantly prolonged with the interventions, from around 5 months to 8.5 to 11 months compared with IFN. The reasons for the slightly lower absolute value for PFS for bevacizumab plus IFN in the CALGB trial are not clear but could be a result of small differences in patient characteristics or differences in the assessment of progression.

Both interventions also appear to have beneficial effects on OS compared with IFN alone, but data are not fully mature from the trials of bevacizumab plus IFN, and interpretation of the data from the sunitinib trial is hampered by the lack of methodological detail.

All three trials were conducted predominantly in patients with metastatic clear cell carcinoma with MSKCC risk factors suggestive of a favourable or intermediate prognosis and who had undergone a previous nephrectomy. Whether these results can be extrapolated to other groups of patients with RCC (e.g., people diagnosed with non-clear cell RCC or defined as having a poor prognosis according to the MSKCC criteria) is unclear.

Data on adverse events suggest that sunitinib is not associated with a greater frequency of adverse events than IFN alone, although the adverse event profile is different and there is some emerging concern about risk of cardiovascular events (European Medicines Agency, 2008; Wu *et al*, 2008). There may be a higher degree of toxicity associated with the combination of bevacizumab and IFN compared with IFN alone.

As there is no head-to-head comparison data available for bevacizumab plus IFN *vs* sunitinib, we performed an indirect comparison which provides evidence that sunitinib may be more efficacious than bevacizumab plus IFN in this patient group at preventing progression. Findings here are consistent with a recently published systematic review, which included an adjusted comparison of the effects of treatment with sunitinib and treatment with bevacizumab plus IFN on PFS, for metastatic RCC. (Mills *et al*, 2009) Mills *et al* report sunitinib to be more effective (HR 0.75, 95% CI 0.60–0.93, *P*=0.001); however, the researchers applied a fairly simple analytical approach. We chose not to perform an indirect comparison of the interventions for OS data due to the lack of mature OS data and the uncertainty surrounding post-study medication use.

There is some debate within the clinical literature as to the relative merits of OS and PFS, as primary end points in oncology trials. It would be unethical to suggest that trial participants be denied additional treatment once trial medication has failed or the benefits of a treatment have been shown. However, the administration of such treatment severely reduces the value of OS estimates obtained from these trials. There may also be bias associated with using PFS as a primary outcome due to the subjective and intermittent nature of progression assessment. Two of the trials included in this review used blinded central review to assess progression, which may remove some of the subjective measurement bias but may introduce complications related to timing of assessment and differences between investigator and independent review. Little is known about the relationship, if any, between PFS and OS in RCC, but it seems likely that the new renal cancer drugs will provide some survival gains.

Interferon was used as the comparator in all three trials. At the time of trial design, IFN was considered the standard therapy for advanced, metastatic RCC. However, the publication of the PERCY Quattro (Negrier *et al*, 2007) trial of immunotherapy in patients with intermediate prognosis suggested that IFN may show no benefit in this patient group at the expense of a high frequency of adverse events. Consequently, in some centres, people with intermediate and poor prognosis are considered to be unsuitable for treatment with IFN and best supportive care becomes their only treatment option.

Using the MSKCC definition, approximately two-thirds of patients (*n*=650) in these three trials were considered to have either intermediate or poor prognosis and could be considered, using alternative definitions, to be unsuitable for treatment with IFN. The extent to which the use of IFN as a treatment comparator in patients who might not benefit from it has influenced the results of these trials is unclear.

There are no published randomised clinical trials of the effectiveness of treatment with sunitinib and treatment with bevacizumab (in combination with IFN) compared with best supportive care in patients who are unsuitable for treatment with IFN. We considered the validity of performing an indirect comparison between IFN and best supportive care to give a better indication of the anticipated benefit of these interventions against best supportive care. However, there are very few trials of IFN *vs* best supportive care (which is variously defined) and those that have been performed do not provide results according to prognostic status. Informal extrapolation of available data suggests that if it is assumed that there is no difference in the relative effectiveness of best supportive care and IFN in this population, even if one assumes a milder adverse event profile with best supportive care, the new interventions should still be considered effective.

In conclusion, there is evidence to suggest that treatment with sunitinib and treatment with bevacizumab plus IFN has clinically relevant and statistically significant advantages over treatment with IFN alone in patients with metastatic RCC in terms of PFS. Beneficial effects on OS have also been reported. In cases where either treatment is considered an option, an indirect comparison of available evidence suggests that treatment with sunitinib has benefits in PFS over treatment with the combination of bevacizumab and IFN.

## Figures and Tables

**Table 1:** Characteristics of included studies

Study	AVOREN (Escudier *et al*, 2008; Bracarda *et al*, 2007; Melichar *et al*, 2007; Escudier *et al*, 2007; Bajetta *et al*, 2008)	CALGB (Rini *et al*, 2008)	Motzer *et al*, 2006(Motzer *et al*, 2007b; Motzer *et al*, 2007c; Figlin *et al*, 2008; Motzer *et al*, 2007a; Motzer *et al*, 2006)
Design	R, DB, PC, phase III, international, multi-centre	R, phase III, international, multi-centre	R, DB, PC, phase III international, multi-centre
*N*	649	732	750
Diagnosis	Metastatic, clear cell RCC	Metastatic, clear cell RCC	Metastatic, clear cell RCC
Prognosis profile according to MSKCC criteria	27 : 56 : 9 (unavailable for 9% of patients)	26 : 64 : 10	38 : 56 : 6
(favourable: intermediate: poor)(%)			
Patients having undergone previous nephrectomy (%)	100	85	90
Intervention and comparator	Bevacizumab plus IFN *vs* placebo plus IFN	bevacizumab plus IFN *vs* IFN	sunitinib *vs* IFN
Outcomes measured	Overall survival^*^, progression-free survival, overall response rate and safety	Overall survival^*^, progression-free survival, objective response rate and safety	Progression-free survival^*^, objective response rate, overall survival, patient reported outcomes and safety

Abbreviations: R= randomised; DB=double blind; PC=placebo controlled; RCC=renal cell carcinoma; IFN=interferon-α. ^*^Primary outcome measure.

**Table 2:** Overall survival

**Analysis**	**Intervention**	**N**	**Median OS (months)**	**95% CI for median OS (months)**	**HR**	**95% CI for HR**	**P-value**
*AVOREN study* (Escudier *et al*, 2008; Bracarda *et al*, 2007; Melichar *et al*, 2007; Escudier *et al*, 2007; Bajetta *et al*, 2008)							
Interim	Bevacizumab plus IFN	327	Not reached	—	0.79	0.62–1.02	0.0670
	Placebo plus IFN	322	19.8	—	—	—	—
							
*Sunitinib vs IFN phase III trial* (Motzer *et al*, 2007b; Motzer *et al*, 2007c; Figlin *et al*, 2008; Motzer *et al*, 2007a; Motzer *et al*, 2006)							
Second interim	Sunitinib	375	Not reached	—	0.65	0.45–0.94	0.02
	IFN	375	Not reached	—			
Final	Sunitinib	375	26.4	23.9–32.9	0.821	0.673–1.001	0.051
	IFN	375	21.8	17.9–26.9			
Censored final^a^	Sunitinib	375	26.4	23.0–32.9	0.808	0.661–0.987	0.0362
	IFN	375	20.0	17.8–26.9			
*Post hoc* final^b^	Sunitinib	193	28.1	19.5–NA	0.647	0.483–0.870	0.0033
	IFN	162	14.1	9.7–21.1			

Abbreviations: CI=confidence interval; HR=hazard ratio; IFN=interferon; NA=not applicable; OS=overall survival.

a *n*=25 patients in the IFN group who crossed over to sunitinib.

b Includes only those patients who received no post-study treatment.

**Table 3:** Progression-free survival

**Analysis**	**Intervention**	**N**	**Median PFS (months)**	**95% CI for median PFS (months)**	**HR**	**95% CI for HR**	**P-value**
*AVOREN study* (Escudier *et al*, 2008; Bracarda *et al*, 2007; Melichar *et al*, 2007; Escudier *et al*, 2007; Bajetta *et al*, 2008)							
Interim	Bevacizumab plus IFN	327	10.2	—	0.63	0.52–0.75	<0.0001
	Placebo plus IFN	322	5.4	—			
							
*CALGB 90206 study* (Rini *et al*, 2008)							
Sixth interim^a^	Bevacizumab plus IFN	369	8.5	7.5–9.7	0.71	0.61–0.83	<0.0001
	IFN	363	5.2	3.1–5.6			
							
*Sunitinib vs IFN phase III trial* (Motzer *et al*, 2007b; Motzer *et al*, 2007c; Figlin *et al*, 2008; Motzer *et al*, 2007a; Motzer *et al*, 2006)							
Second interim	Sunitinib	375	11^b^	10–12	0.42	0.32–0.54	<0.001
	IFN	375	5^b^	4–6			
Interim data cut-off February 2007	Sunitinib	375	11^b^	10.7–13.4	0.538	0.439–0.658	<0000001
	IFN	375	5.1^b^	3.9–5.6			
Censored final^c^	Sunitinib	375	Not reported	Not reported			
	IFN	375					
*Post hoc* final^d^	Sunitinib	193	Not reported	Not reported			
	IFN	162					

Abbreviations: CI=confidence interval; HR=hazard ratio; IFN=interferon; OS=overall survival; PFS=progression-free survival.

a Estimate of HR adjusted for stratification factors – nephrectomy status and MSKCC risk factors.

b Results from independent review.

c *n*=25 patients in the IFN group who crossed over to sunitinib.

d Includes only those patients who received no post-study treatment.

**Table 4:** Progression-free survival: indirect comparison of sunitinib *vs* bevacizumab plus IFN

**Comparison**	**Input data**	**HR (95% CI)**	**P-value**
Sunitinib *vs* IFN	Motzer: HR=0.538; SE[ln(HR)]=0.103	0.538 (0.439, 0.658)	<0.0001^*^
Bevacizumab *vs* IFN	{AVOREN: HR=0.630; SE[ln(HR)]=0.093 CALGB: HR=0.710; SE[ln(HR)]=0.079}	0.676 (0.601, 0.760)	<0.0001^*^
Sunitinib *vs* bevacizumab	[Indirect comparison]	0.796 (0.630, 1.004)	0.0272^*^

Abbreviations: HR=hazard ratio; IFN=interferon.

Based on 100 000 MCMC iterations.^*^One-tailed *P*-values.
